# Relationship between Acceptance of Insects as an Alternative to Meat and Willingness to Consume Insect-Based Food—A Study on a Representative Sample of the Polish Population

**DOI:** 10.3390/foods10102420

**Published:** 2021-10-13

**Authors:** Klaudia Modlinska, Dominika Adamczyk, Dominika Maison, Katarzyna Goncikowska, Wojciech Pisula

**Affiliations:** 1 Institute of Psychology, Polish Academy of Science, Jaracza 1, 00-378 Warsaw, Poland; katarzyna.goncikowska@gmail.com (K.G.); wojciech.pisula@wp.pl (W.P.); 2 Faculty of Psychology, University of Warsaw, Stawki 5/7, 00-183 Warsaw, Poland; dominika.adamczyk@psych.uw.edu.pl (D.A.); dominika@psych.uw.edu.pl (D.M.)

**Keywords:** insects as food, food neophobia, variety-seeking, environmental concerns, disgust, consumer studies, eating, food choices, sustainable meat alternative

## Abstract

Despite their nutritional and ecological potential, insect-based food is rarely accepted by consumers. There may be a discrepancy between the consumers’ understanding of the need to reduce meat consumption and their personal food preferences. Our goal was to investigate the relationship between the acceptance of insects as a meat substitute, the willingness to buy and consume insect-based food, and the underlying factors. The study was conducted on a representative sample of the Polish population, and as in previous studies, our results showed that men who are more familiar with entomophagy pay more attention to the environmental impact of their food choices, are convenience-orientated and are more willing to accept insects as a meat substitute. However, people with higher levels of food neophobia and disgust sensitivity and lower levels of variety-seeking tendency are less willing to consume insects. Our study showed that the acceptance of insects as an alternative to meat (general perspective) does not translate into a willingness to buy and eat them (individual perspective). Consumers who declare their acceptance of insects as a meat substitute might not be willing to purchase insects for their consumption.

## 1. Introduction

Although growing evidence suggests that reducing meat consumption is inevitable due to the environmental impact and ethical issues linked to meat production, the consumption of meat substitutes among Western consumers is still low (e.g., [1]). The strongest objections are voiced with regard to insects (e.g., [2,3]), which could be a sustainable source of protein and other essential nutrients [3,4,5,6]. Even in several developing countries such as Kenya, where inhabitants are culturally accustomed to consuming certain types of insects, attempts at introducing novel insect-based food often meet with resistance [7]. Moreover, the consumption of insects is in decline in countries where it used to be the norm [8,9].

To date, several factors have been identified as barriers to introducing insects into the daily diet. The main variable is food neophobia: the reluctance to eat novel food [10]. Persons with high levels of this psychological trait are among those most resistant to the idea of ingesting insects (e.g., [11,12,13]). These findings demonstrate the importance of familiarity with a particular product for its acceptance as food [14,15,16,17]. Prior exposure to the consumption of insects creates an indicator of cultural appropriateness of this kind of food [18,19,20] and translates into greater willingness to ingest it [21].

Another significant factor linked to the reluctance to consume insects is disgust [22], which results from associating insects with dirt and decay [23]. Disgust is often assumed to be one of the major arguments for rejecting insects, as indicated by consumers ([24,25,26,27], but [27,28]).

On the other hand, some influential factors have been identified as promoters of insect consumption. An intrinsic desire for variety (the “variety-seeking tendency”) is recognised as an essential factor influencing consumer food choice [26]. Its significance was also shown in studies on insect consumption where a high variety-seeking tendency correlated with a greater willingness to eat insect-based food [27,29,30,31,32].

Considering the environmental and ethical advantages of insect production, it is also possible that factors influencing lower meat consumption may play a role in the willingness to ingest insects. A set of personally held ideas may influence decisions linked to the choice of food [33]. Individuals who are concerned about the degradation of the natural environment, are more likely to accept novel kinds of food if they are persuaded of their beneficial impact on nature (see [21,26,34,35]).

Health issues are another essential factor that influences dietary choices [36]. Insects may be considered a valuable source of animal nutrients, which are insufficiently present in vegetarian diets, such as amino acids or vitamin B12 [3]. Knowledge about the health benefits of this type of meat substitute may influence the willingness to incorporate it into one’s diet (cf. [37,38]).

Analysing all these factors may allow us to create a specific profile of a potential consumer of insect-based foodstuffs. To date, there is a shortage of studies presenting comprehensive findings drawn from representative samples, which hinders the application of research findings to marketing strategies. The first consumer profile of the population willing to adopt insects as food was presented by Verbeke [21]. The study, which was conducted on a representative sample of the Belgian population, indicated that factors such as gender, familiarity, food neophobia, convenience, and pro-environmental food choice motives, as well as attitudes to meat and future meat consumption intentions, play a significant role in predicting the level of acceptance of insects as an alternative to meat. The study showed that the target group for insect-based foodstuffs is younger males without strong attitudes towards meat who are open to trying novel foods (low food neophobia) and interested in the environmental impact of their food choices. The likelihood that such consumers are ready to adopt insects as a meat substitute was estimated at 75%. The author suggests that this study, conducted in Belgium, represents a typical Western consumer profile, which may be true, as most of the identified factors were also found in studies conducted in other countries (e.g., [17,39]).

Other studies examined factors influencing the acceptance of insect-based food, and although they were conducted on large populations, none of them included nationwide samples. A study by Hartmann and colleagues [40] revealed that low scores on the food neophobia scale, positive taste expectations, high scores on social acceptance, and experiences eating insects were significant predictors of a willingness to eat insects in both Germany (*n* = 502) and China (*n* = 443). The authors underlined the importance of familiarity with products containing insects for reducing neophobic reactions and negative attitudes towards this kind of food.

Among Hungarian consumers (*n* = 400), the most willing to accept insect-based food were those who seek novel food options and intend to reduce their meat intake [41]. Findings from studies conducted on Italian consumers showed, similarly to a study by Verbeke [21], that the readiness to accept insects was higher among males than females and among younger consumers than older [39]. Although knowledge about environmental factors of insect production positively affected the level of acceptance of insect-based products among professionals and students, there was no sustainable daily efforts in this respect [39]. In Switzerland (*n* = 379), several predictors of the willingness to consume insects were identified, such as convenience, the discernibility of insects in food, expected health benefits, need for familiarity, food neophobia, food technology neophobia, perceived health benefits of meat, as well as gender and experience [17]. Again, younger males were identified as the most willing to try insects, but the authors suggest that it may result from a higher rate of familiarity with this type of food in this group. On the other hand, the attachment to meat was a negative predictor of insect acceptance. Contrary to other studies, food neophobia, although important, was not found to be the key predictor of the willingness to consume insects, and it was closely correlated with familiarity. A survey among Chinese consumers (*n* = 614) depicted factors such as entomophobia, disgust, knowledge about insects, and demographic variables such as age, household size, household income, and region of residence [42]. A recent study conducted on Italian and Danish consumers (*n* = 300), the group most willing to adopt insects as food was identified among the so-called rational food consumers (i.e., people who seek information about products, use shopping lists or look for new ways to prepare food) [43].

Despite numerous attempts to create a profile of a prospective consumer of insect-based food, sales and consumption remain low. We hypothesize that it may be due to an overlap of two different dimensions related to insects’ consumption, hindering a closer understanding of these phenomena (cf. [19]). First, the need to reduce meat production and consumption creates the market for its substitutes. People aware of environmental and health issues may be more in favor of the idea of meat substitutes (for review [44]; see also [26]). Therefore, it seems that those who paying attention to the environmental impact of food choices may be a suitable target for insect-based foodstuffs. It is likely that asked about acceptance of insect-based foods they would voice a positive attitude. Second, there are multiple psychological factors responsible for willingness to consume novel and unusual food [11,12,13,14,15,24,25,30,31], which may hinder the willingness to purchase and consume insects. Yet, it is possible that acceptance of the idea and a willingness to purchase and consume insects are not correlated, and different factors predict both phenomena. This notion is based on the suggestion of Sogari and colleagues [14] that providing knowledge about the environmental and nutritional benefits of insect consumption is not likely to influence consumers (see also [13]). However, some previous studies (cf. [2,21,45,46,47]) seem to be based on the idea that the combined analysis of these two areas allows the creation of a profile of a customer willing to buy and eat insects. It is likely that the discrepancy between these two matters may require different educational and marketing strategies.

Therefore, the goal of our study was to investigate the relationship between the acceptance of insects as a meat substitute and the willingness to buy and consume insect-based foodstuffs. In our analyses, we incorporated psychological and demographic variables which had been found to be relevant in previous studies. Regression models were used to conduct the analyses.

## 2. Materials and Methods

The study was conducted from November to December 2020 in an online panel specializing in social studies with the use of a Computer-Assisted Web Interview (CAWI). All participants gave their informed consent after reading detailed information about the experiment. They were asked to click on a link to the study if they agreed to take part. The study had been approved by the Research Ethics Committee at the Faculty of Psychology, University of Warsaw.

To verify the hypothesis about the discrepancy between the acceptance of insects as a meat substitute and the willingness to buy and consume insect-based foodstuffs, we planned a study involving separate analyses of both phenomena followed by an investigation of the relation between them. The study was accompanied by a series of questionnaires and questions measuring levels of factors potentially related to both areas of study. These factors were chosen based on previous studies to allow for the comparison of results.

### 2.1. Participants

A nationwide representative random quota sample of a total of 1096 people was selected from among the online panel (55% women; 45% men), aged 16–78 years (M = 44, SD = 15.15). The sample structure corresponded to the structure of the Polish population (the quotas were selected based on the distribution of gender, age, education, and the size of the city). The descriptive statistics of the sample are presented in Table 1.

### 2.2. Questionnaires and Scales

#### 2.2.1. Attitude to Adopting Insects as a Meat Substitute (MS)

Consumer readiness to adopt insects as a meat substitute was measured by asking the participants to provide their response to one item: ‘‘Insects could replace meat in our diet’’. The participants assessed the statement on a 5-point Likert scale (1-totally disagree, 5-totally agree). Contrary to the study by Verbeke [21], we did not provide prior information about the benefits of eating insects (in the original study, the participants were told “[insects] are a good source of high-value proteins; their production requires little space; their feed conversion is efficient; therefore, eating insects provides benefits in terms of sustainability’’). We decided to proceed this way because informing participants about the benefits of eating insects and their production could influence their assessment of whether insects could replace meat in their diet [35,48].

#### 2.2.2. Acceptance of Food with Insect-Derived Ingredients (EA)

The participants’ acceptance of food with insect-derived ingredients was measured by the Entomophagy Attitude Questionnaire (EAQ) [12]. This tool comprised 10 questions, and participants assessed them on a 7-point Likert scale (from 1-“completely disagree” to 7-“completely agree”). Statements included, “I would be disgusted to eat any dish with insects”; “I think it is fine to give insect-based feed to fish that are farmed for human consumption”; and “I’d be curious to taste a dish with insects if cooked well”. The items were merged into one entomophagy attitude (EA) score. The internal consistency of the Polish version of the scale in our study measured using Cronbach’s alpha was estimated at 0.88.

#### 2.2.3. Familiarity with Insect Consumption (Fam)

The participants were also asked four questions regarding their experience and awareness of insect consumption. According to Verbeke [21], the first two questions concerned the awareness of entomophagy as a phenomenon (“Have you heard that in other countries people eat insects on a daily basis?” (yes/no) and “Have you heard that in our country you can buy edible insects in the store?” (yes/ no). Because numerous studies (e.g., [17]) show that previous insect consumption is a strong predictor of willingness to consume insects, the subsequent questions were related to the experience with eating insect-based food (“Have you ever eaten insects or food containing them?”—No, never; I am not sure; Yes, once; Yes, more than once) and preparing this kind of food (“Have you ever tried to make a dish with insects or with ingredients from insects?”—No, never; Yes, a few times; Yes, many times). The information recorded through these questions was summed up, and this variable was used as a proxy for self-reported familiarity (Fam) with the notion of entomophagy. 

#### 2.2.4. Attitudes towards Health Characteristics of Food (FHealth)

Consumer attitudes towards the health characteristics of food were measured using three items: ‘‘I pay attention to how the food I choose affect my health’’; ‘‘I know which food is healthy and which is not’’; and ‘‘When shopping, I choose products that have labels indicating their health benefits’’. Each item was scored on a 7-point scale (1–totally disagree; 7–totally agree). The three items were merged into one food health (FHealth) interest score—Cronbach’s alpha = 0.81.

#### 2.2.5. Convenience Orientation in Relation to Food (FConv)

Convenience orientation in relation to food was measured using one item—“I don’t care what I eat, satisfying my hunger is the most important thing”. The item was scored on a 7-point scale (1—totally disagree, 7—totally agree), and it indicated an interest in choice based on food convenience (FConv). 

#### 2.2.6. Consumers’ Attention to the Environmental (FEnv) Impact of Their Food Choices 

Consumers’ attention to the environmental impact of their food choices was measured using a method similar to the method used in the study by Verbeke [21], but using three items: “When I buy food, I try to pay attention to how its production affects the environment”; “I try to avoid food products whose production is harmful to the environment”; and “I am interested in how food production affects the environment”. The first item, following the study by Verbeke [21], was based on Roberts [46], and the other two were additional. All three items were scored on a 7-point scale (1—totally disagree; 7—totally agree). These three items were summed up, and treated as indicators of attention to the environmental impact of the participants’ food choices (Cronbach’s alpha = 0.86).

#### 2.2.7. Food Neophobia (FN)

Food neophobia was measured by means of the Food Neophobia Scale (FNS) [49], which is used to measure the willingness to try new foods. The scale consists of 10 statements: five positive (indicative of neophilic attitudes) and five negative (indicative of neophobic attitudes). For example: “I don’t trust new food’’; and “At dinner parties, I will try a new food”. Participants assessed the statements on a 7-point scale (1—strongly disagree; 7—strongly agree. The internal consistency of the Polish version of the scale in our study measured using Cronbach’s alpha was estimated at 0.84.

#### 2.2.8. General Neophobia (GN)

The general level of neophobia was measured with the General Neophobia Scale (GNS). GNS was developed together with the Food Neophobia Scale by Pilner and Hobden [49]. The scale comprises eight items evaluated on a 7-point Likert scale (1—strongly disagree; 7—strongly agree). Examples of statements include “I am afraid of the unknown”; and “Whenever I am on vacation, I can’t wait to get home”. The internal consistency of the Polish version of the scale in our study measured using Cronbach’s alpha was estimated at 0.91.

#### 2.2.9. Disgust Sensitivity (DS)

Individual differences in sensitivity to disgust were measured with the Disgust Scale-Revised [50]. The scale consists of 22 items: the first 13 are binary “true” or “false” questions, and the rest assess how disgusting a given experience seems on a scale from 0 (“not disgusting at all”) to 2 (“very disgusting”). Example items include “Even if I was hungry, I would not drink a bowl of my favorite soup if it had been stirred by a used but thoroughly washed flyswatter”; and “Seeing a cockroach in someone else’s house doesn’t bother me”. The internal consistency of the Polish version of the scales in our study measured using Cronbach’s alpha was estimated at 0.83.

#### 2.2.10. Variety Seeking (VS)

The individual level of variety-seeking tendency in the context of food choice was measured with the Variety Seeking Tendency Scale (VARSEEK-Scale) [29]. The respondents assessed eight statements on a five-point Likert scale (1—completely disagree; 5—completely agree). Examples of statements include “I prefer to eat food products I am used to”; and “When I eat out, I like to try the most unusual items, even if I am not sure I would like them”. The internal consistency of the Polish version of the scale in our study measured using Cronbach’s alpha was estimated at 0.91.

#### 2.2.11. Experience with Traveling for Business (TravB) and for Pleasure (TravP)

The participants were asked about their experience with traveling abroad: “How often did you travel to other countries for business purposes in the past year?”; and “How often did you travel to other countries in the past year for leisure?” For both questions, the participants chose their answer from the following options: Not at all, less than once a year, 1–3 times a year, approximately every quarter, almost every month, more than once a month.

#### 2.2.12. Socio-Demographic Characteristics

In addition, we recorded various socio-demographic characteristics such as age, gender, education (primary/secondary or higher) and place of residence (village, town with a population of <10,000, city with a population of between 100,000 and 500,000, city with a population of >500,000). The participants were also asked about their dietary preferences, choosing from one of three options: vegetarian (people who do not eat meat, including fish and seafood, but who eat other animal products), vegan (people who do not eat meat or any animal products, e.g., eggs, milk, honey), and omnivorous (people who do not limit their consumption of meat or animal products).

### 2.3. Statistical Modeling

Due to the different construction of data related to the questions about the acceptance of insects as a meat substitute and the willingness to consume insect-based foodstuffs, we employed two regression models to investigate both issues as described in more detail below. The analysis of these separate problems and their predictors was followed by the investigation of the correlation between them using Pearson’s r statistic.

#### 2.3.1. Descriptive Analyses

The data were analyzed using a combination of descriptive techniques (means, frequencies, percentages) using SPSS 26.0.0.1. The Student’s *t* test and analysis of variance (ANOVA) were employed to assess the differences in the attitude towards insects in different demographic groups.

#### 2.3.2. Binary Logistic Regression

The logistic regression model was used to assess the acceptability of the idea of insects as meat substitutes. As in Verbeke [21], the dependent variable was analyzed as a discreet answer (no/yes). 77.1% of the respondents were against, and 22.9% were in favor of the idea.

Regression equations:

Readiness to adopt insects as a meat substitute = b_0_ + b_1_ × gender + b_2_ × age + b_3_ × edu + b_4_ × diet + b_5_ × FN+ b_6_ × GN + b_7_ × VS + b_8_ × DS + b_9_ × Fam+ b_10_ × FConv + b_11_ × FHealth + b_12_ × FEnv

Gender, education, and diet were specified as dummy variables. Regression coefficients were estimated using maximum likelihood estimation and are presented with Wald χ^2^ statistics and as odds ratios.

#### 2.3.3. Multiple Regression

The multiple regression model was employed to assess the acceptance of food with insect-derived ingredients. Assumptions of linearity, normality, homoscedasticity, and multicollinearity were checked.

Regression equations:

Acceptance of food with insect-derived ingredients = b_0_ + b_1_ x age + b_2_ × FN+ b_3_ × GN + b_4_ × VS + b_5_ × DS + b_6_ × Fam+ b_7_ × FConv + b_8_ × FHealth + b_9_ × FEnv

Regression analyses were conducted using the Jasp v. 0.14.1 [51] statistical software developed by the University of Amsterdam, Netherlands.

## 3. Results

### 3.1. Descriptive Statistics

Eighty-eight percent of the participants indicated that they had heard that insects were eaten in other countries. Of these, 38% stated that they had heard that it was possible to buy edible insects in Poland. Only 7% had had experience eating insects, and 4% had tried to prepare them.

Self-reported familiarity with insect consumption was linked to the frequency of travel. A higher level of familiarity was associated with a higher frequency of traveling both for business (r = 0.261, *p* < 0.001) and for leisure (r = 0.201, *p* < 0.001) (Table 2). Familiarity with eating insects also correlated with the importance of food convenience (r = 0.160, *p* < 0.001). No correlation was observed between familiarity and the other variables. 

However, significant correlations were found between the acceptance of food with insect-derived ingredients (EA) and the food neophobia, general neophobia, and disgust sensitivity scales. Higher acceptance of food with insects was associated with lower levels of food neophobia (r = −0.362, *p* < 0.001), general neophobia (r = −0.162, *p* < 0.001) and disgust sensitivity (r = −0.066, *p* = 0.029). On the other hand, the correlation with variety-seeking tendency was positive, and a higher level of acceptance of insect-based foods was associated with a higher level of VS (r = 0.328, *p* < 0.001). 

Attitudes towards the health characteristics of food positively correlated with attention to the environmental impact of food choice (r = 0.748; *p* < 0.001) and negatively correlated with a convenience-oriented attitude (r = −0.182, *p* < 0.001). 

Attitudes towards health, environmental and conventional aspects of food correlated with age. Older age was linked to more positive attitudes towards health (r = 0.098, *p* = 0.001) and environmental aspects of food (r = 0.106, *p* < 0.001), whereas younger age was associated with more positive attitudes towards convenience (r = −0.159, *p* < 0.001). 

Mean scores for the FN, GN, DS, and VS scales are presented in Table 1. As assumed by Pliner and Hobden [38], a positive correlation between FN and GN was observed (r = 0.390, *p* < 0.01). A higher level of food neophobia was associated with a higher level of general neophobia. A lower level of food neophobia was linked to a higher level of variety-seeking tendency (r = −0.735, *p* < 0.01) (Table 2). No significant correlation between food neophobia and disgust sensitivity was found. Although sensitivity to disgust did not correlate with food neophobia and variety seeking, we observed a correlation between sensitivity to disgust and general neophobia. A higher level of disgust sensitivity was associated with a higher level of general neophobia (r = 0.123, *p* < 0.001). The level of general neophobia was also negatively correlated with variety seeking (r = −0.248, *p* < 0.001). These correlations and their directions are in line with the theoretical assumptions indicating negative correlation of general neophobia with sensation seeking and positive correlation between general neophobia and disgust [7].

Some gender differences were also observed. The Student’s *t*-test showed that female participants paid more attention to both the health (M = 15.05 for females vs. M = 14.20 for males; *t* = 3.86; *p* < 0.001) and environmental (M = 14.03 for females vs. M = 13.00 for males, *t* = 4.21; *p* < 0.001) aspects of food. However, females tended to be less familiar with the concept of entomophagy (M = 5.11) than the male participants (M = 5.31), *t* = −2.70; *p* = 0.007. No significant differences were found for the other explanatory variables, nor did education level have any significant impact on differences in the other explanatory variables.

Few respondents (12%) reported limiting meat or animal products: 9% described themselves as vegetarians and 3% as vegans. These results are in line with the data obtained in previous surveys over a similar period (e.g., in the 2019 representative sample survey 8.4% of the population identified as vegetarians and vegans [52]).

### 3.2. Insects as a Meat Substitute-Binary Logistic Regression Results

Logistic binary regression was run to predict acceptance of the idea of insects as meat substitutes from FN, GN, VS, DS, FHealth, FEnv, Fam, Fconv, gender, edu, diet, and age. Results indicated that there was a significant effect of gender, familiarity with insect consumption (Fam), attention to the environmental impact of food choices (FEnv), and convenience-orientated approach to food choices (FConv)—χ^2^ (1083) = 99.581, *p* < 0.001; R2 = 0.132. Table 3 presents detailed results of the binary logistic regression model.

The analysis showed a significant effect of gender but no effect of age or educational level. Males were 1.8 times more likely to admit that insects can be a substitute for meat. Diet had no effect. Vegetarians, vegans, and omnivores accepted insects as a meat substitute at the same level.

Participants who were familiar with the concept of entomophagy were 1.3 times more likely to accept insects as a meat substitute compared to those who were not familiar or who were less familiar with the idea. Similarly, attention to the environmental impact of food choices was responsible for a 1.3-times higher likelihood of accepting insects as meat alternatives. There was also an effect observed in the case of the convenience orientation. Persons who pay attention to their food choices were 1.1-times more likely to accept the idea of insects as a meat substitute.

The most influential factor in the statistic was familiarity (Wald χ^2^ = 19.661), followed by attention to environmental impact (Wald χ^2^ = 14.583).

None of the psychometric properties (food neophobia, general neophobia, sensitivity to disgust, variety-seeking tendency) had any effect on accepting the idea of insects as meat substitutes.

### 3.3. Acceptance of Food with Insect-Derived Ingredients-Multiple Regression Results

Multiple regression was run to predict willingness to accept insects as food with insect-derived ingredients from FN, GN, VS, DS, FHealth, FEnv, Fam, Fconv, and age. All assumptions of linearity, normality, homoscedasticity, and multicollinearity were found to be met. The multiple regression model significantly predicted the level of willingness to accept insects as food (F(8,1087) = 20.650, *p* < 0.001; R2 = 0.146). Regression coefficients and standard errors are presented in Table 4.

Food neophobia and sensitivity to disgust negatively predicted the willingness to accept insects as food. Higher scores on those variables translated into a lower level of willingness to accept insects. On the other hand, the variety-seeking tendency positively predicted the dependent variable. Persons with higher levels of variety seeking were more willing to accept insects as food.

Food neophobia was found to be the most influential factor accounting for 4% of the variance (controlling for other variables). It was followed by the variety-seeking tendency, which accounted for 1%.

Familiarity with the notion of entomophagy (Fam), attention to the environmental impact of food choices (FEnv), and convenience orientation of food choices (FConv) had no effect on the acceptance of food with insect-derived ingredients.

There was no significant correlation between the two dependent variables used in the regression models: acceptance of the idea of insect-based foods as meat substitutes and willingness to accept food with insect-derived ingredients (r = 0.020; *p* = 0.509).

## 4. Discussion

The data gathered in our study showed that there is a significant discrepancy between the acceptance of the idea of using insects as a meat substitute and the actual willingness to accept food with insect-derived ingredients. Our analyses indicated that both variables are not correlated and that they differ in their explanatory factors. As in Verbeke’s study [21], in our study, acceptance of insects as an alternative to meat was predicted by the familiarity with the concept of entomophagy, attention to the environmental impact of food choices, and convenience orientation for food choices. Also, men were more favorable to this idea than women. However, there was no effect of food neophobia or diet preferences. On the other hand, acceptance of insects as food was predicted by food neophobia, disgust sensitivity, and variety-seeking tendency. As the predictors for both issues are similar to those identified in the previous studies, it is reasonable to believe that the differences are not due to the confounding factors or specificity of the population but rather stem from the distinctive nature of the two areas.

A possible explanation for the differences between findings from our study and those from the study by Verbeke [21] is the construction of dependent variables. In our analyses, we aimed to separate, on the one hand, the effect of awareness of the environmental need for decreasing meat production and finding substitutes and on the other hand personal attitudes to the consumption of insects. We hypothesized that there may be a difference between these two attitudes. In the study by Verbeke [21], the question ‘‘I would be prepared to eat insects as a substitute for meat’’ comprised both problems, which may be why his findings combine results from our separate questions. It may be the case also in other studies (e.g., 47, 53).

This explanation was confirmed by our analyses of the explanatory variables. In our study, familiarity and attention to the environmental impact of food predicted the level of acceptance of the general idea of insects as a meat substitute but not the willingness to eat them (self-behavior declaration). It seems that participants who are familiar with the possibility of incorporating insects into their diets and sensitive to ecological issues may be in favor of the sustainable advantages of insects as meat alternatives (cf. [13,14,48]), but they may be opposed to the idea that they themselves should eat insects. As many previous studies have shown (e.g., [11,12,13,20,21,27]), people are generally opposed to eating insects, and the main factors responsible for this attitude are food neophobia and sensitivity to disgust. This seems irrelevant to the familiarity with the idea of eating insects somewhere in other countries.

Our findings clearly showed that familiarity was also a complex factor. Most people were aware that insects are eaten in other countries, and this correlated with the frequency of traveling abroad, but very few of them had eaten insects or prepared meals with them. Thus, their familiarity was based on a general concept rather than on experience, and it did not explain the willingness to try insect-based foods (cf. [26]).

Contrary to the findings by Verbeke [21], we did not find any effect on the attitude to meat on either the willingness to eat food with insect-derived ingredients or the acceptance of insects as meat substitutes. Verbeke [21] asked general questions about meat consumption and its advantages, and he excluded vegetarians and vegans from the study. This approach limited conclusions to the omnivorous population and eliminated the groups often involved in searching for meat substitutes (e.g., [53,54]). By asking about the respondents’ behaviour (i.e., about a specific diet), we were able to control for this factor. However, we found that diet had no effect on attitudes to insects. The result shows that vegans and vegetarians were no more willing to accept insects as a meat substitute than omnivores were. It is probably because vegans and vegetarians often perceive insects as animals, and they have moral doubts about their farming and consumption (unpublished qualitative data).

As in previous studies (e.g., [21,55]), attitudes towards healthy characteristics of food had no impact on either of the dependent variables. It seems that insects are not perceived as a healthy alternative to meat and that participants might not be aware of their nutritional properties. The association of insects with dirt and contamination [21] may hinder their acceptance as a healthy food source. 

## 5. Conclusions

In view of the above, we may conclude that the acceptance of the idea of insects as a meat substitute is not synonymous with the acceptance of the actual consumption of insects. Consumers who declared their acceptance of this kind of alternative to meat may be unwilling to buy insects for their own consumption. In line with findings from previous studies, the highest likelihood of consuming insect-based foods has been observed in persons with a low level of food neophobia and low disgust sensitivity but with a high level of variety-seeking tendency. Moreover, although men are more familiar with the notion of entomophagy and more willing to accept the idea of insects as a meat substitute, they are not more likely than women to ingest them.

It appeared that educating people about sustainable properties of insect-based foodstuffs and aiming marketing strategies in this feature is not sufficient to convince consumers to buy and eat insects. Insects seem to be a proper meat alternative only in theory. A similar effect appears to be evoked by familiarity, which is the factor often identified as a predictor of the willingness to consume insects. Again, it is likely that familiarity with the idea of eating insects increases the acceptance of the basic concept and not a willingness to try real insect-based food.

## Figures and Tables

**Table 1:** Descriptive statistics of dependent and explanatory variables (*n* = 1096).

Variable	Label	Mean (SD)
*Dependent variables*		
Attitude to adopt insects as a meat substitute (1 = no, 2 = yes)	MS	1.23 (0.42)
Acceptance of food with insect-derived ingredients (min = 1, max = 7)	EA	3.57 (1.16)
*Explanatory variables*		
Familiarity with insect consumption (min = 4, max = 12)	Fam	5.20 (1.2)
Attitude towards the health characteristics of food (min = 3, max = 21)	FHealth	14.66 (3.66)
Convenience orientation in relation to food (min = 1, max = 7)	FConv	3.21 (1.74)
Attention to the environmental impact of food choices (min = 3, max = 21)	FEnv	13.57 (4.06)
Food neophobia (min = 10, max = 68)	FN	34.22 (9.41)
General neophobia (min = 8, max = 56)	GN	30.75 (9.35)
Disgust Sensitivity (min = 2, max = 24)	DS	12.72 (2.99)
Variety-Seeking (min = 8, max = 56)	VS	38.54 (7.95)
Travelling frequency—business (min = 1, max = 6)	TravB	1.67 (1.08)
Travelling frequency—pleasure (min = 1, max = 6)	TravP	2.21 (1.11)
Gender (1 = female, 2 = male)	Gender	1.45 (0.50)
Age (years)	Age	43.94 (15.15)
Diet (1 = vegetarian, 2 = vegan, 3 = none)	Diet	2.79 (0.60)
Education (1 = primary or secondary, 2 = higher)	Edu	1.44 (0.49)
Place of residence (1–4)	Place	2.09 (1.03)

Min and max values present the level of scores reached by participants in the present study.

**Table 2:** Bivariate Pearson correlations between metric explanatory variables (*n* = 1096).

	EA	Fam	FHealth	FConv	FEnv	FN	GN	DS	VS	TravB	TravP	Age
EA	1											
Fam	−0.017	1										
FHealth	−0.017	−0.034	1									
FConv	−0.019	0.160 **	−0.182 **	1								
FEnv	−0.005	0.035	0.748 **	−0.094 **	1							
FN	−0.362 **	0.016	−0.006	−0.010	−0.029	1						
GN	−0.162 **	0.023	−0.014	−0.018	−0.019	0.390 **	1					
DS	−0.066 *	−0.010	0.019	0.035	−0.018	0.020	0.123 **	1				
VS	0.328 **	0.008	0.026	−0.006	0.046	−0.735 **	−0.248 **	0.001	1			
TravB	0.028	0.261 **	0.081 **	0.211 **	0.120 **	−0.012	−0.012	−0.001	0.020	1		
TravP	0.014	0.201 **	0.116 **	0.093 **	0.137 **	−0.016	−0.002	0.028	0.028	0.532 **	1	
Age	0.014	−0.046	0.098 **	−0.159 **	0.106 **	−0.068 *	−0.016	−0.008	0.050	−0.174 **	−0.114 **	1

* *p* < 0.05; ** *p* < 0.01.

**Table 3:** Results of binary logistic regression model explaining consumers’ acceptance of the idea of insects as meat substitute.

					Wald Test		
	Estimate	Standard Error	Odds Ratio	z	Wald Statistic	df	*p*
(Intercept)	−5.663	1.103	0.003	−5.135	26.367	1	<0.001
FEnv	0.119	0.031	1.127	3.819	14.583	1	<0.001
Fam	0.272	0.061	1.313	4.434	19.661	1	<0.001
FConv	0.107	0.044	1.112	2.415	5.831	1	0.016
FN	0.019	0.013	1.019	1.543	2.381	1	0.123
FHealth	−0.030	0.034	0.970	−0.880	0.775	1	0.379
GN	0.000	0.009	1.000	0.027	7.365 × 10^−4^	1	0.978
VS	0.015	0.014	1.015	1.052	1.107	1	0.293
DS	0.016	0.025	1.016	0.627	0.394	1	0.530
gender (2)	0.579	0.156	1.784	3.703	13.713	1	<0.001
edu_rek (2)	0.030	0.153	1.030	0.194	0.038	1	0.846
diet (2)	0.673	0.424	1.960	1.587	2.518	1	0.113
diet (3)	−0.382	0.246	0.682	−1.552	2.410	1	0.121

**Table 4:** Results of multiple regression model explaining consumers’ willingness to eat foods with insects.

Model		Unstandardized	Standard Error	Standardized	*t*	*p*
H_0_	(Intercet)	3.575	0.035		103.242	<0.001
H_1_	(Intercet)	4.378	0.459		9.537	<0.001
	FN	−0.032	0.005	−0.258	−5.895	<0.001
	VS	0.021	0.006	0.141	3.379	<0.001
	DS	−0.026	0.011	−0.066	−2.356	0.019
	FHealth	−0.005	0.013	−0.016	−0.387	0.699
	FEnv	0.001	0.012	0.005	0.111	0.912
	Fam	−0.026	0.026	−0.027	−0.973	0.331
	FConv	0.012	0.019	0.019	0.648	0.517
	age	−0.001	0.002	−0.017	−0.619	0.536
	GN	5.285 × 10^−4^	0.004	0.004	0.139	0.890

## Data Availability

The data presented in this study are available on request from the corresponding author. Although consumer data have been anonymised, data are not publicly available.
